# GenomeGems: evaluation of genetic variability from deep sequencing data

**DOI:** 10.1186/1756-0500-5-338

**Published:** 2012-07-02

**Authors:** Sharon Ben-Zvi, Adi Givati, Noam Shomron

**Affiliations:** 1 Department of Biomedical Engineering, The Iby and Aladar Fleischman Faculty of Engineering, Tel-Aviv University, Tel Aviv, Israel; 2 Department of Cell and Developmental Biology, Sackler Faculty of Medicine, Tel-Aviv University, Tel Aviv, Israel

**Keywords:** Deep sequencing, Next generation sequencing, Software, Genetic analysis, Data interpretation, Variance calling

## Abstract

**Background:**

Detection of disease-causing mutations using Deep Sequencing technologies possesses great challenges. In particular, organizing the great amount of sequences generated so that mutations, which might possibly be biologically relevant, are easily identified is a difficult task. Yet, for this assignment only limited automatic accessible tools exist.

**Findings:**

We developed GenomeGems to gap this need by enabling the user to view and compare Single Nucleotide Polymorphisms (SNPs) from multiple datasets and to load the data onto the UCSC Genome Browser for an expanded and familiar visualization. As such, via automatic, clear and accessible presentation of processed Deep Sequencing data, our tool aims to facilitate ranking of genomic SNP calling. GenomeGems runs on a local Personal Computer (PC) and is freely available at http://www.tau.ac.il/~nshomron/GenomeGems.

**Conclusions:**

GenomeGems enables researchers to identify potential disease-causing SNPs in an efficient manner. This enables rapid turnover of information and leads to further experimental SNP validation. The tool allows the user to compare and visualize SNPs from multiple experiments and to easily load SNP data onto the UCSC Genome browser for further detailed information.

## Findings

The sequencing of the human genome was the highlight of many years of international laborious efforts. Since 2004, new technologies termed “Deep Sequencing” or “Next-Generation Sequencing” have been developed to reduce timelines and costs of subsequent re-sequencing of additional human genomes [1,2]. These methods changed genome sequencing approaches, made genome sequencing extremely accessible, and opened new fields in biomedical investigation [3,4]. Basically, Deep Sequencing methods allow enormous amounts of short DNA fragments to be read simultaneously [5]. One of the most common applications is the discovery of genetic variation between healthy and diseased individuals [6,7]. In particular, the emergence of Deep Sequencing technologies has dramatically boosted whole genome sequencing and re-sequencing [8,9]. This advancement possesses computational challenges for base calling, read alignment, genome assembly, mutation detection as well as data visualization [3,10-12]. Currently, a variety of software tools are available for analyzing Deep Sequencing data. These range from alignment of the nucleotide reads to a reference genome, base-calling, polymorphism detection, genome browsing and annotation [13]. A subset of these tools, those that provide a better evaluation and visualization of Deep Sequencing data is presented in Table 1 and 2. Of particular importance, the final stage of the Deep Sequencing data interpretation pipeline, where genetic variance is identified and evaluated, requires distinct attention. The potential candidate mutations are screened and ranked for their relevance for further investigation. Hence, they are a critical gateway of the investigators to the disease-causing mutation.

**Table 1:** Comparison of some of the currently avaibale tools for data interpretaion and analysis

**Tool**	**Operating system**	**Language**	**Database**	**Alignment format**	**Data file formats**
**ABC**[14]	Win, Linux, and OS X	JAVA	Multiple sequence alignments and data typically associated with alignments	NA	NA
**EagleView**[15]	Win, Linux, Mac OS X	NA	Next-generation sequencing data	NA	ACE format (commonly used by genome assembly programs), READS, EGI, MAP
**LookSeq**[16]	An AJAX based web viewer. Requires a standard web browser	Perl, AJAX	Illumina genome analyzer sequencing data	1. Both CIGAR and new sequencing technology alignment data	NA
				2. SAM/BAM format of SAM tools	
**Magic Viewer**[17]	Win	Java	Next-generation sequencing data	NA	SAM format- enables an easy conversion of various input file formats, including PSL, MAQ, Bowtie, SOAP, ZOOM
**Tablet**[18]	Windows, OS X, Linux, Solaris	Java	ACE, AFG, MAQ and SOAP assembly formats. Also 454 and Solexa data	NA	NA
***GenomeGems ***	Win	MATLAB	Next-generation sequencing data, analyzed by MAQ, Variant SNP Classifier, and SNVMix in, a pre-determined ‘.txt’ format	Especially MAQ, but also Variant SNP Classifier and SNVMix	Reads '.txt’ file format with columns separated by tab

Abbreviations: *NA*, not available; *MAQ*, Mapping and Assembly with Quality; *SOAP*, Short Oligonucleotide Analysis Package; *ACE*, Archive Compression Extension; *AFG*, Auxiliary File Generator; *EGI*, Embedded Gateway Interface; *SNVMix*, Small Nucleotide Variants; *CIGAR*, Compact Idiosyncratic Gapped Alignment Report.

**Table 2:** **A comparison of the Visualization Capabilities and Data Integration of the different tools currently available with those of *GenomeGems***

**Tool**	**View**	**Data integration**
ABC [14]	Three distinct display modes:	NA
	1. A very low resolution- histogram	
	2. Intermediate resolutions- a ‘Wiggly Plot’	
	3. Very high resolution - the user may view the sequence data directly.	
EagleView [15]	Compact with zooming capability.	Genome features (exon, intron, etc.), Polymorphism data (e.g. SNP), 454 flowgram trace, Illumina four color raw signals.
	Pinpoint view of: base quality, technology-specific sequence trace, read ID and strand.	
LookSeq [16]	1. A resolution from the level of a whole chromosome to the level of individual bases.	LookSeq can visualize read alignments and some basic properties as separate “tracks”:
	2. There are options to view genome coverage, GC content, and annotations to the reference sequence.	1. Sequence annotation
		2. Coverage
		3. GC contents
		This information is taken from the alignment databases as well as some auxiliary files.
Magic Viewer [17]	The short read image can be zoomed to any resolution, from a whole chromosome to individual bases at any desired level.	NA
	Also displays auxiliary information: read ID, location, base quality, read length and orientation.	
Tablet [18]	The main display provides a view of a single contig at a time, with reads aligned against their consensus sequence.	NA
GenomeGems	Five separate analysis methods are available:	GenomeGems integrates well with the UCSC Genome Browser, for the purpose of visualization of SNPs, in addition to the analysis and visualization in the actual tool.
	1. Data Table - displays the data supplied by the user and analyzes the percentage of mutant reads, in spreadsheet format, enabling analysis within the tool in addition to fast export to Excel.	UCSC custom tracks supply additional data calculated by UCSC such as: context of the SNP – CDS or intron, and the properties of the changed amino acid – polarity, acidity and hydropathy.
	2. Sample Comparison - displays a bar graph presenting the frequency of each SNP in the investigated samples, according to a threshold value.	
	3. SNP-View - displays a table containing the numbers of samples that include each SNP in a specific chromosome.	
	4. Translation of the input file into a PgSNP file format for a later visualization in the UCSC, as a UCSC Custom Track.	
	5. Additional Information- suggests additional external links for further investigation and annotation of specific SNPs and of the impact of amino acid changes on human proteins.	

Abbreviations: *NA*, Not available; *PgSNP*, Personal Genome SNP; *CDS*, Coding Sequence; *UCSC*, University of California Santa Cruz.

Several tools exist to facilitate the data interpretation stage, each focusing on a different aspect of the analysis: EagleView [15], for example, is compatible with a variety of operating systems and supports visualization of Deep Sequencing derived genome assemblies. However, this software, freely available on the internet, is not suitable for the most up-to-date sequencing technologies (such as ABI/SOLID or Helicos). LookSeq [16], an AJAX based web viewer was developed to visualize the multiple layers of information which includes large data sets of aligned sequence reads, produced by Deep Sequencing, and enable the user to visualize the information at different levels of resolution. This tool uses Illumina Genome Analyzer/HiSeq 2000 data as input though lacks the ability to visualize large sequenced regions such as an entire human chromosome due to significant memory demands. MagicViewer [17], a freely available application based on an independent operating system implementation, provides annotation facilities for Single Nucleotide Polymorphisms (SNPs) without extending annotations for Insertion-Deletions (Indels). In addition, it lacks features of conducting comparisons among various samples. ABC [14], a Java based viewer for exploration of data associated with alignments displays quantitative data (such as sequence similarity) and annotation data (such as location of genes and repeats), simultaneously. ABC does not function as a genome-wide browser, but is suitable for comparative sequence analysis. Finally, Tablet [18] displays the data as highly packed views allowing instant navigation to any region of interest. Compatible with a variety of operating systems, Tablet requires large memory storage therefore has limited use on a Personal Computer (PC).

Our tool, termed *GenomeGems*, was developed in order to provide systematic means to reduce inconsistency in selecting which genetic variances or mutations should be further investigated. We developed a unique interface which includes analysis and visualization (via the widely used UCSC Genome Browser) leading to prioritization of data generated by Deep Sequencing runs. One way to facilitate variance calling from genetic sequences is putting them in context with other sequenced samples [19]. Therefore, one of *GenomeGems’* strong features lies within its ability to compare, analyze and visualize a large number of samples, simultaneously. Using tables and graphs on a PC workstation, both Microsoft Excel and the UCSC Genome Browser are directly linked to the interpreted information. While some tasks carried out by *GenomeGems* can be achieved by other standalone tools, such as the ‘R package’ or also partially by Microsoft Excel, *GenomeGems* is a suite of applications which makes it easier to perform a combination of tasks accessible for end users of non-computational background. This tool comes to facilitate genomic research via multiple-processing and accessible presentation of Deep Sequencing data for variance calling, in order to assist rapid turnover of information leading to further experimental mutation detection. Since SNPs are the most prevalent genetic modification among individuals [20]*GenomeGems* currently focuses on these variations.

## Rationale

**Figure 1:**
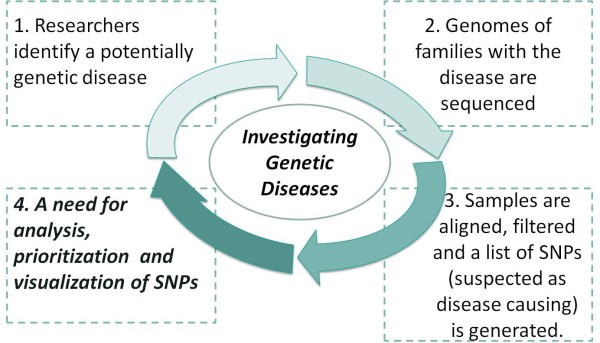
**An illustration of a common research process done when investigating a potential genetic disease.** This interdisciplinary process normally involves researchers from three distinct disciplines: bio-medical discipline, Deep Sequencing laboratory, and bioinformatics discipline. (1) Researchers from the bio-medical discipline identify a potentially genetic disease. (2) Genomes of afflicted individuals or of whole families are sequenced using Deep Sequencing technology. (3) The sequences acquired are compared with a consensus sequence in order to find SNPs. (4) A list of SNPs and Indels is consequently generated and is filtered. (5) Finally a list of SNPs and Indels is produced which possibly contains the disease causing mutation. The list usually contains either novel or clinically associated SNPs (6) These lists are submitted to the researchers in the bio-medical discipline, for further analysis.

During the investigation of disease-causing genetic mutations using Deep Sequencing methods, there are multiple steps along the analysis pipeline (schematically shown in Figure 1). First, biomedical researches select a disease and try to identify the underlying genetic causes behind it. Consequently, genomes of affected individuals, or of whole families, are sequenced using Deep Sequencing machines. The data acquired is compared with a consensus sequence using bioinformatics alignment tools such as MAQ [21], and is assessed and annotated for the presence of variants using tools such as Variant Classifier and SNVMix [22]. At this point, a list of SNPs (and Indels) is accordingly generated and is filtered for high confidence values. The list of SNPs produced presumably contains the disease-causing mutation. These lists are usually separated into two based on whether they are novel or clinically associated SNPs by comparing to comprehensive databases such as dbSNP [23]. These files are extremely valuable as they lead to further analysis and confirmation on a larger set of samples. Yet, at this point these records frequently contain hundreds of SNPs in text format, and researchers are faced with the often tedious task of filtering the candidates in search for the disease-causing mutation. The task of filtering the list can be carried out using tabular lists (such as Microsoft Excel tables) and using a variety of freely available online databases and tools such as: dbSNP [23], PolyPhen-2 [24], ConSurf [25], and others. These tools contain data of previously reported SNPs [23] and of the amino acid change such SNPs are expected to generate. If this analysis is carried out manually it becomes tedious, time consuming, repetitive, and prone to inaccuracy. *GenomeGems* is directed specifically for the purpose of providing researchers with a simple tool for sorting, analyzing, prioritizing and visualizing the SNPs provided by data acquired by Deep Sequencing experiments (as long as the input file adheres to the *GenomeGems’* format). While several features of our software can be performed by other standalone tools, such as the ‘R package’ or also partially by Microsoft Excel, *GenomeGems* makes it easier to carry out a combination of tasks accessible for end users of non-computational background.

## Methods

**Figure 2:**
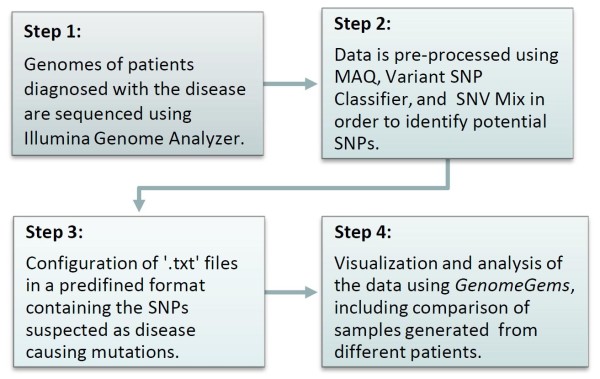
A flow chart describing the process performed on a sample data.

The key design feature underlying *GenomeGems’* application is to facilitate the final steps of Deep Sequencing data analysis via organizing and allowing accessible presentation of the data, thus leading to a rapid shift to the next step of experimental mutation detection. *GenomeGems* was validated using Deep Sequencing data generated in the Genome High-Throughput Sequencing Laboratory at Tel-Aviv University on the Illumina Genome Analyzer apparatus. A sample processing pipeline is presented in Figure 2. *GenomeGems* was developed using MATLAB functions and MATLAB’s Graphic User Interface (GUI) tools. It functions as a stand-alone application on a Windows workstation with ActiveX Control and “MCR Ver 7.10” installation required on the users’ workstations. These software necessitates minimal hardware, memory usage and installation time. The user can easily download these software installation packages from the *GenomeGems* website. *GenomeGems* was carefully designed paying particular attention to the requirements of the investigators in this genomic field. Algorithms were developed for a simple comparison using graphs and tables of data produced from a number of samples, and for a wide and detailed visualization of the Deep Sequencing pre-processed data. *GenomeGems* integrates well with the University of California Santa Cruz (UCSC) Genome Browser for the purpose of SNP visualization within investigated chromosomes. This function is made possible by development of a platform for the conversion of pre-processed input data to a Personal Genome SNP data format (PgSNP) [26], which can be viewed and further analyzed using the UCSC Genome Browser. Furthermore, *GenomeGems* suggests additional useful external databases for further clinical SNP investigation.

### Application

**Figure 3:**
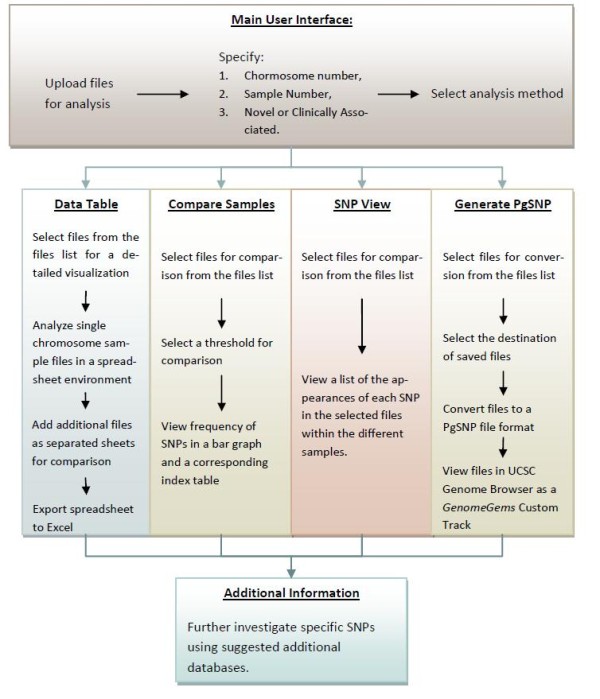
**An illustration of the different analysis functions of GenomeGems in a schematic workflow.** The user uploads the SNP files in the pre-determined format and chooses the form of analysis required: translation to a PG-SNP file format for UCSC visualization, visualization via data table, sample comparison via bar graph or table. In addition, more information about investigated SNPs can be obtained by using the suggested links to external databases.

A basic implementation of *GenomeGems* has been developed, which enables the user to analyze and visualize the input data according to the flow chart in Figure 3.

### Main user interface

**Figure 4:**
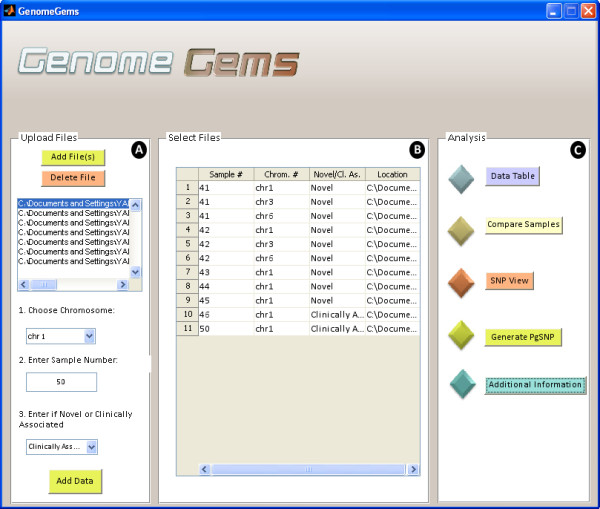
**The GenomeGems main user interface contains three distinct panels, (A) Upload FIles, (B) Select Files and (C) Analysis.** The user may upload an unlimited number of samples and chromosomes that will later be available from each of the analysis tools. More analysis functions may be added to the Analysis panel in the future, as the tool is built in a modular design.

The main user interface contains three distinct panels as seen in Figure 4: (A) Upload Files, (B) Select Files, and (C) Analysis. The Upload Files panel contains a list into which the user uploads the input files, selects a chromosome on which the analysis will be performed, specifies the sample number and specifies whether the data is of novel or clinically associated SNPs. The user may upload multiple files containing multiple samples, but each file must be of one single sample. The user may also choose multiple chromosomes on which the later analysis will be performed.

The selected files, with a specified sample number, chromosome number, novel or clinically associated and location appear in the ‘Select Files’ panel (marked as B) as a list. This list of files must include all of the files that are required for the later analysis. At any stage the user may return to the main user interface in order to add more files to be available for analysis. The ‘Analysis’ panel (marked as C) contains the different functions available for analysis. At the moment, the tool contains five options for analysis: Data Table, Compare Samples, SNP View, Generate PgSNP, and Additional Information. In the future, additional forms of analysis will be added to this panel, as the tool is built in a modular form, allowing for further expansion.

## Input file format

**Figure 5:**
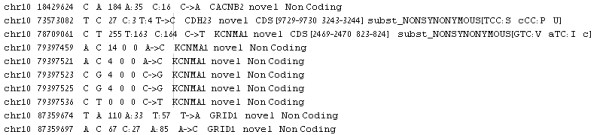
**Example of the input file format required for GenomeGems.** The file must contain data from one single sample, and must not contain a heading line. The file may contain one single chromosome or all chromosomes, but in both cases the user must specify the chromosome for analysis. The data in the file must be separated into columns using tabs, and must contain the first 7 columns: chromosome number, SNP position, consensus nucleotide, SNP nucleotide, score of the SNP, number of reads for each nucleotide, as shown in the figure. The file may include any additional data in the following columns, also separated by tabs.

Users input a list of SNPs after analysis by MAQ (or other software) in a pre-determined format. The files must be in “.txt” format and columns are separated by a tab. The files must contain the following data (in this specific order): Chromosome number, SNP Position, Consensus Nucleotide, SNP nucleotide, Score of the SNP, Number of Reads of each nucleotide. If any information is missing the user is directed to use “0”. Other optional information that can be submitted: Gene Name, SNP Novel/ Known, CDS (Coding Sequence)/Non-Coding, Synonymous/Non-Synonymous, Amino Acid Replacement, SNP ID for known SNPs and so on. See Figure 5 for an example of a sample file. Data input is supplied by uploading the files that are to be analyzed, and choosing the chromosomes relevant for each file. This list of files and chromosomes is saved, and is later accessed throughout the employment of the tool.

## Data table

**Figure 6:**
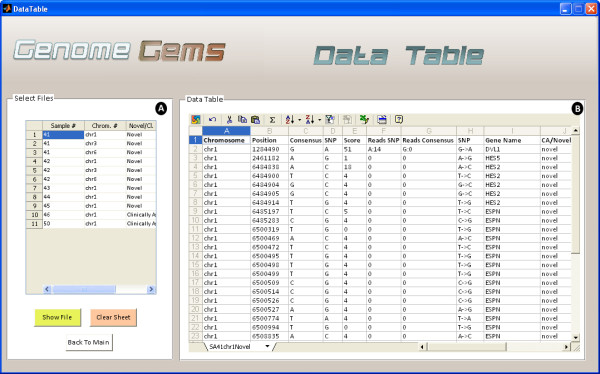
**The Data Table analysis interface enables the user to (A) select the files for viewing, one at a time and (B) view the data in a clear and familiar MS Spreadsheet environment, allowing easy export to Excel.** Multiple files may be shown as separate sheets.

The Data Table user interface (shown in Figure 6) enables analysis of the data uploaded by the user inside the actual tool in addition to fast export to Excel using Microsoft Office Spreadsheet ActiveX Control component. The data table visualizes the information associated with the chromosome that was selected by the user. It allows presenting a number of samples and chromosomes simultaneously as different sheets. The data table shows all of the information that was supplied by the user in a tabular fashion. In addition, the percentage of the mutant reads is displayed for an easier determination of SNP Homozygosity or Heterozygosity.

## Sample comparison

**Figure 7:**
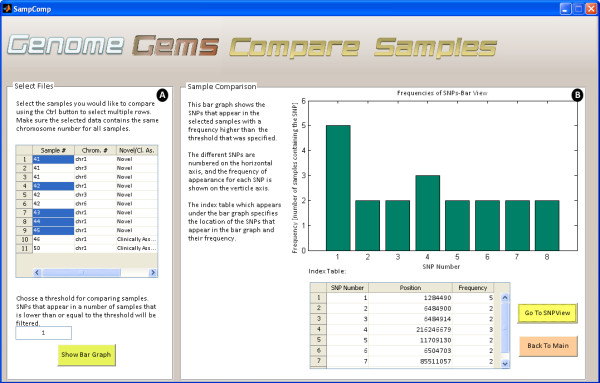
The Compare Samples interface allows the user to (A) select files for comparison and choose a threshold for minimal SNP frequency and (B) view the results in a bar graph and a corresponding index table.

When searching for a disease causing mutation, multiple samples are sequenced from a population which is either related or is diagnosed with the specific disease. In case several samples are uploaded into the *GenomeGems*, the user may compare samples and calculate the frequency of appearance of each SNP in the different samples. This information is displayed as a bar graph showing the frequencies of each SNP which surpass the threshold value selected formerly by the user, along with a corresponding table which serves as an index (as shown in Figure 7).

## SNP view

**Figure 8:**
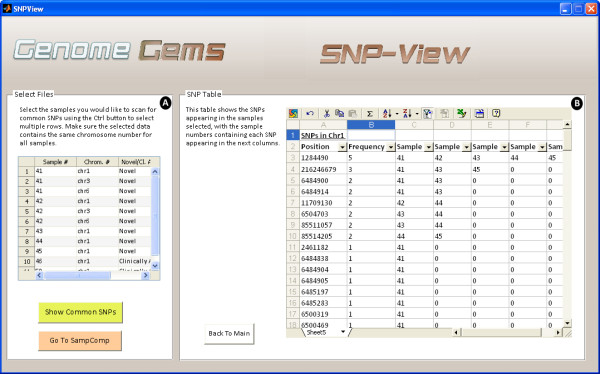
**The SNP-View interface allows the user to (A) select sample files for comparison containing the same chromosome number and (B) view a list of SNPs appearing in the selected samples, in the specified chromosome, with a list of the samples in which each SNP appears.** The list may be easily exported to Excel for further analysis.

Upon selection of desired files for analysis, the ‘SNP View’ interface (shown in Figure 8) displays a table containing the sample numbers that include each SNP in the specific chromosome defined formerly. This data may be useful for further analysis by the users, and can be easily exported to Microsoft Excel.

## PGSNP file format in the UCSC Genome Browser

**Figure 9:**
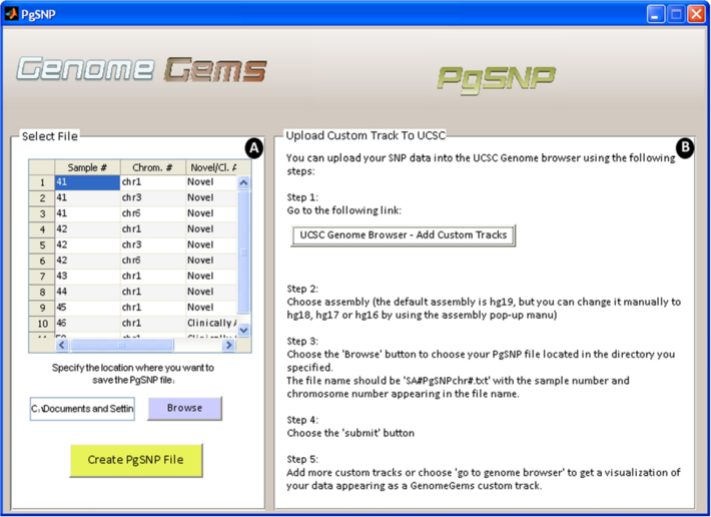
The PgSNP interface allows the user to (A) choose a file for conversion to PgSNP format and specify the location where the file will be saved, and (B) instructs the user how to upload the file to UCSC as a Custom Track in five simple steps.

Many tools have been developed to examine the structure and function of the human gene set. For this purpose, genome browsers from the NCBI and UCSC have been designed. *GenomeGems* is designed to be compatible with the UCSC Genome Browser, created by the University of California Santa Cruz, as it is commonly used to analyze genetic information. It provides a graphical display of related genes that can be organized based on specific criteria such as expression levels, proximity in genome, protein similarity, and Gene Ontology (GO) [14,15]. By using *GenomeGems*, users can automatically convert the original format of data into a PgSNP format that can be viewed conveniently in UCSC Genome Browser using UCSC’s custom tracks feature (shown in Figure 9).

## Custom tracks in the UCSC

**Figure 10:**
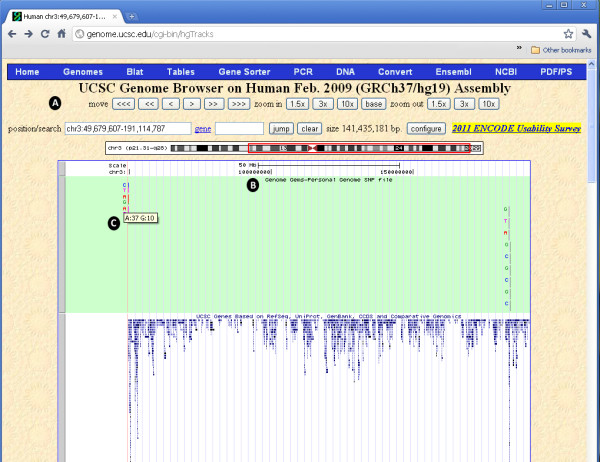
**The UCSC Genome Browser allows the user to view the data uploaded into GenomeGems as a custom track.** (**A**) The user can manipulate the view with options of move, zoom in, and zoom out, (**B**) the custom track appears at the top of the screen and can be set to hide, dense, squish, pack and full, and (**C**) when the user moves the mouse control over the specific SNP, the frequency of each allele is shown.

**Figure 11:**
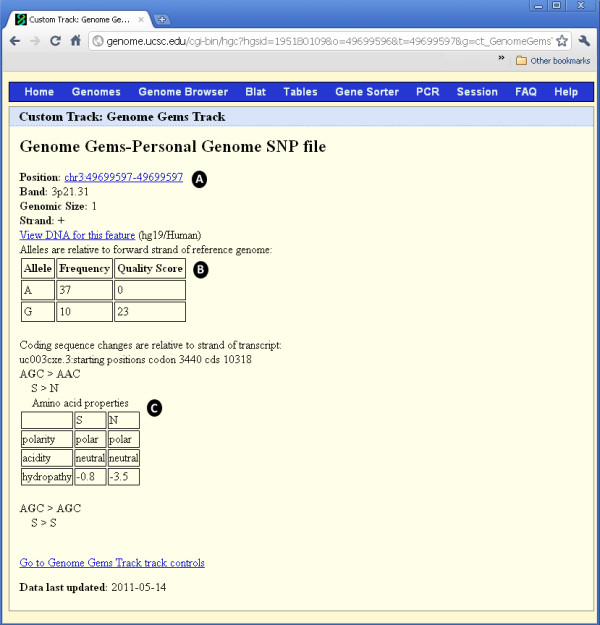
**When the user chooses one of the SNPs appearing in the UCSC visualization interface, a new window opens containing (A) the position of the SNP, in addition to band, genomic size and strand, (B) the frequency and quality score for each allele, and (C) the properties of the changed and original amino acids: polarity, acidity and hydropathy.** Notice the alleles are relative to forward strand of reference genome, and the coding sequence changes are relative to the strand of transcript.

Custom tracks enable research scientists using the UCSC Genome Browser to visualize their own results or annotation tracks alongside standard annotation tracks. This simple tool may be used to display locations of SNPs as well as other information regarding each SNP [[27],[28]]. *GenomeGems* uses an algorithm for generating PgSNP files from the original data files, which can then be uploaded as a custom track in the UCSC Genome Browser. A display of the SNPs uploaded by the user is consequently created and supplementary information supplied by UCSC can be viewed. The additional information supplied by UCSC is the context of the SNP – CDS or Intron, and the properties of the changed amino acid–polarity, acidity and hydropathy, as seen in Figures 10 and 11.

## Additional information

**Figure 12:**
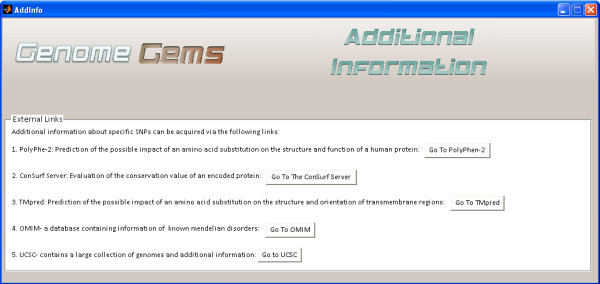
The Additional Information interface enables quick transfer to suggested additional databases for further analysis of SNPs.

For further investigation and annotation of specific SNPs and of the impacts of amino acid changes encoded by the mutant gene on a human protein, *GenomeGems* suggests additional external useful links: Polymorphism Phenotyping v2( PolyPhen-2) [24] , Server of the Identification of Functional Regions in Proteins (ConSurf Server) [25], Prediction of Transmembrane Regions and Orientation (TMpred), Online Mendalian Inheritance in Man (OMIM) [29] and University of California Santa Cruz (UCSC) [30], (see Figure 12). The ‘Additional Information’ interface will be updated in the next versions of *GenomeGems* to enable direct referral to a specific entry in the databases, based on the user’s SNP selection.

## Application summary

*GenomeGems* enables researchers to identify potential disease-causing SNPs in an efficient manner. *GenomeGems’* main advantages are its: (i) ability to integrate data from several Deep Sequencing runs on a standard PC; (ii) assimilation with the UCSC Genome Browser and Microsoft Excel; (iii) applicability for any Deep Sequencing data (given the correct input file format) (iv) power to compare and analyze a large number of samples. *GenomeGems'* main virtues allow: (i) reducing variability in selecting which mutations should be further investigated; (ii) facilitating genomic research via clear and accessible presentation of processed Deep Sequencing data; (iii) assisting rapid turnover of information and a quick lead to further experimental mutation detection.

## GenomeGems facilitates genomic research

Behind the implementation of *GenomeGems* lies our main objective of facilitating genomic research by processing Deep Sequencing data in a comprehensive and accessible fashion. This enables rapid turnover of information and leads to further experimental SNP validation. The tool allows the user to compare and visualize SNPs from multiple experiments and to easily load SNP data onto the UCSC Genome browser for further detailed information.

## Further developments

In addition to the currently implemented features of *GenomeGems*, development of additional elements for further analysis is underway. *GenomeGems* was designed using a modular approach, enabling easy extension of its capabilities. Continuous dialogue with potential end-users of *GenomeGems,* and constant search for improvements, ensures that more advanced features will be added to the current implementation. A few examples are listed below.

1. Indel Analysis

At the moment, the tool does not support data files containing indels. An extension of the tool will include indel analysis and an algorithm for determining whether an indel causes the appearance of a nonsense mutation in the sequence analyzed.

2. Full Genome Analysis

At the moment *GenomeGems* enables analysis of a single chromosome specified by the user. In the next version of *GenomeGems* we intend to enable full genome analysis and full genome comparison between samples.

3. Additional Visualization Capabilities

The current version of *GenomeGems* enables SNP visualization by means of UCSC’s Custom Tracks. In subsequent versions a convenient visualization within the application and without the need to connect to the Internet will be included.

4. Further mutation Analysis

The current version of *GenomeGems* lacks an independent feature for prediction of the impacts amino acid substitutions (caused by SNPs) on the structure and function of human proteins. Instead, external free tools providing this information are suggested. In subsequent versions this feature will be included as an integrated function of *GenomeGems*.

## Availability of the software and system requirements

Project Name: *GenomeGems.*

Project Home Page: http://xwww.tau.ac.il/~nshomron/GenomeGems.

Operating System: Microsoft Windows.

Programming Language: MATLAB 2009.

Other Requirements: installation of an ActiveX Control and “MCR Ver 7.10” on the users' workstations.

## Abbreviations

SNPs: Single Nucleotide Polymorphisms; Indels: Insertion-Deletions; MAQ: Mapping and Assembly with Quality; UCSC: University of California Santa Cruz; NCBI: National Center of Biotechnology Information; SNVMix: Small Nucleotide Variants; PgSNP: Personal Genome SNP; CDS: Coding Sequence; NA: Not available; SOAP: Short Oligonucleotide Analysis Package; ACE: Archive Compression Extension; AFG: Auxiliary File Generator; EGI: Embedded Gateway Interface; SNVMix: Small Nucleotide Variants; CIGAR: Compact Idiosyncratic Gapped Alignment Report.

## Competing interests

The authors declare no competing financial interest.

## Authors’ contributions

NS conceived the need for the software, SB, AG and NS designed the tool, SB and AG wrote the software, SB, AG and NS wrote the paper. All authors read and approved the final manuscript.
